# Traditional ecological knowledge for monitoring *Anaphalis javanica* (DC.) Sch.Bip. (Asteraceae) in Bromo Tengger Semeru National Park, Indonesia

**DOI:** 10.1007/s10661-024-12869-8

**Published:** 2024-07-09

**Authors:** Denni Susanto, Prasetyo Nugroho, Shinya Numata

**Affiliations:** 1 Bachelor of Applied Science in Forest Management, Vocational College of Universitas Gadjah Mada, Yogyakarta, Indonesia; 2 Department of Tourism Science, Tokyo Metropolitan University, Tokyo, Japan

**Keywords:** Traditional ecological knowledge, Indigenous communities, *Anaphalis javanica*, National parks, Conservation, Population

## Abstract

Traditional ecological knowledge (TEK) possessed by indigenous communities offers a vital source of information regarding the status and threats faced by species. *Anaphalis javanica* (DC.) Sch.Bip. commonly known as Javanese edelweiss is a culturally significant species in Indonesia’s mountainous regions, yet conservation efforts for it remain limited. We utilized TEK to assess the status and threats to the Javanese edelweiss in Bromo Tengger Semeru National Park, Indonesia. The research aims to assess the distribution, abundance, population trends, and threats faced by Javanese edelweiss using TEK. We interviewed 641 individuals from seven Tengger villages near the park to gather data on the distribution, status, and threats facing the Javanese edelweiss, contributing to conservation efforts. Data were analyzed using generalized linear mixed models and ordinal logistic regression. TEK regarding the Javanese edelweiss was extensive, as 96.3% of respondents recognized the plant. A majority (73.2%) had observed Javanese edelweiss in the wild, yet a significant portion (85%) perceived it as “very rare” or “rare.” Furthermore, 50% of respondents reported a decline in its population. Variations in sighting frequency, perceived abundance, and population trends across different locations provide crucial insights for identifying priority areas and shaping community-based conservation strategies for Javanese edelweiss. This research underscores the importance of integrating TEK into conservation planning, emphasizing community involvement for effective and sustainable biodiversity conservation.

**Supplementary Information:**

The online version contains supplementary material available at 10.1007/s10661-024-12869-8.

## Introduction

One of the main causes of biodiversity loss is hunting and/or resource extraction by indigenous human communities is one of the main causes of biodiversity loss, particularly in Southeast Asia (Sodhi et al., 2004; Zhang et al., 2020). Conversely, traditional ecological knowledge (TEK) held by indigenous communities can also play an important role in biodiversity conservation, both within Indonesian national parks (Susanti & Zuhud, 2019) and in areas outside of protected zones (Hernández-Morcillo et al., 2014; Leiper et al., 2018). TEK, the indigenous knowledge passed down through generations via cultural practices, includes an understanding of the relationships among living beings, including humans, and their environment (Berkes, 1993; Berkes et al., 2000; Hernández-Morcillo et al., 2014; Sinthumule & Mashau, 2020).

Understanding population status, distribution, and threats is key to effective biodiversity conservation management (Cheng et al., 2021). An evidence-based approach is critical for tracking changes in protected plant populations (Collen et al., 2013) and designing and implementing effective conservation plans (White et al., 2013). However, these approaches can often be time-consuming and costly (Chambers et al., 2014; Cheng et al., 2021). Furthermore, some threatened species are cryptic and difficult to study, challenging traditional methods (Cheng et al., 2021). This can lead to a dearth of data on certain species’ population status, distribution, and threats (Archer et al., 2020; Willcox et al., 2019). Therefore, researchers sometimes utilize knowledge from local communities that coexist with these species and have extensive experience observing them in their natural habitats (Azzurro et al., 2019; Davis & Wagner, 2003).

Over recent decades, TEK has gained recognition as an invaluable resource in natural resource assessment and management, comparable to conventional research methods (Azzurro, 2018; Nazareth et al., 2022; Rochet et al., 2008). It has been employed in various contexts, such as assessing the status of threatened species, understanding species distribution over extended timeframes (Ceríaco et al., 2011; Drew, 2005; Joa et al., 2018; Phuthego & Chanda, 2004; Truong, 2022; Turvey et al., 2015; Wilkinson & Van Duc, 2017), predicting changes in populations (Berkes et al., 2000; Furusawa et al., 2014; Peñaherrera-Palmaa et al., 2018; Benner et al., 2021), and identifying threats to certain species (Abram et al., 2015; Caballero-Serrano et al., 2019; Nash et al., 2016; Tamou et al., 2018). Studying TEK offers a deeper ecological understanding of individual species and ecological changes over time (Carter & Nielsen, 2011). TEK is advantageous for bridging research or management gaps through long-term local observations (Gilchrist et al., 2005; Hall & Close, 2007) and for generating large-scale data for conservation planning at reduced costs (Aswani & Lauer, 2006; Anadón et al., 2009). However, its direct application in practical conservation planning remains limited (Archer et al., 2020).

Some potential limitations and biases may arise in the collection of TEK data, including varying levels of knowledge among respondents due to sociodemographic differences (Beaudreau & Levin, 2014; Iniesta-Arandia et al., 2014; Papworth et al., 2009). Nevertheless, these biases can be reduced through appropriate data collection methodologies that account for regional sociodemographic characteristics (Archer et al., 2020). Moreover, carefully designed TEK evaluations can yield reliable information useful for managing data-poor natural resources (Thurstan et al., 2016; Peñaherrera-Palmaa et al., 2018).

The Javanese edelweiss (*Anaphalis javanica* (DC.) Sch.Bip. (Asteraceae)) is an endemic species to the mountains of Java, Indonesia (Oo et al., 2022; Roziaty & Wijaya, 2019). It is a pioneer species in young volcanic soil and can thrive in barren lands (Hernawan et al., 2020; Lathifah et al., 2019). Presently, the prevalence of Javanese edelweiss in the Bromo Tengger Semeru National Park (BTSNP) is declining (Ade et al., 2021), and it has been reported as absent along the Mount Semeru climbing route (Amalia et al., 2019). Notably, Javanese edelweiss is a protected plant in Indonesia (regulation no. P.106/MENLHK/SETJEN/KUM.1/12/2018). The main threats in the BTSNP include illegal harvesting by the community for use as souvenirs (Rahma et al., 2022), utilization in traditional activities by local people (Ade et al., 2021; Ifa et al., 2019), and the impacts of climate change (Maghiar et al., 2021). Therefore, comprehensive primary ecological data on its distribution, abundance, trends, and threats are crucial for monitoring and conservation (Tuohy et al., 2022; Willcox et al., 2019).

This study explores the application of TEK to ascertain the distribution, abundance, population changes, and threats to the Javanese edelweiss (*Anaphalis javanica* (DC.) Sch.Bip.) in BTSNP. We investigated TEK among indigenous communities to assess the status and threats to Javanese edelweiss in BTSNP. We also explored the willingness of indigenous peoples to participate in species monitoring activities, aiming to gain a deeper understanding of their attitudes toward conservation. This study highlights the importance of integrating TEK into conservation planning and management. By respecting and incorporating local community perspectives, conservation initiatives can achieve greater effectiveness and sustainability over the long term. Our research complements previous studies by highlighting the yet underexplored role of TEK from indigenous peoples, particularly those closely connected to national parks, in providing valuable ecological information about threatened plants. Additionally, our findings offer insights for conservation practices both in the study area and in broader contexts where TEK plays a critical role in biodiversity conservation.

## Materials and methods

### Study area

**Fig. 1:**
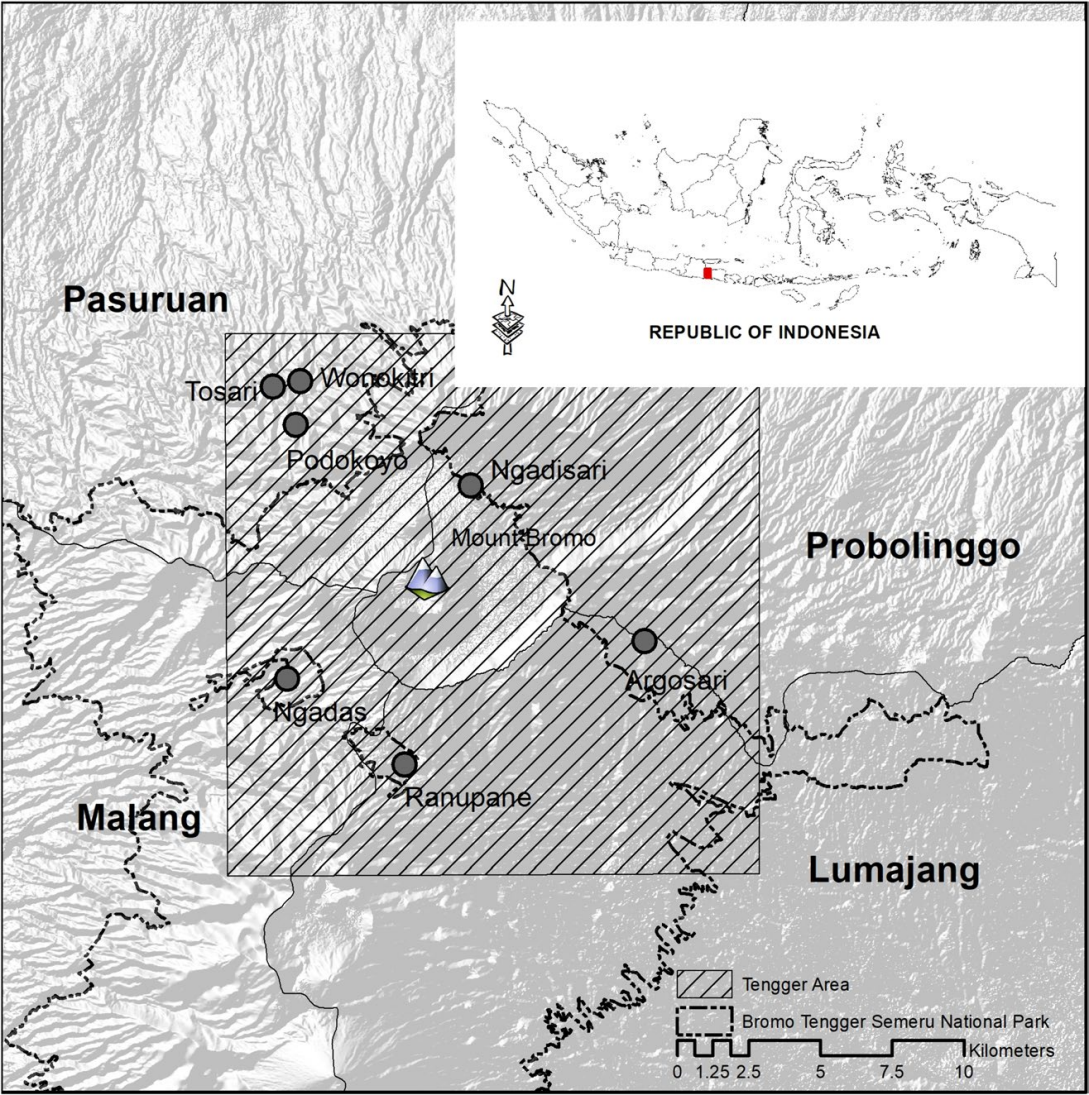
Study areas in East Java Province, including the districts of Pasuruan, Probolinggo, Malang, and Lumajang

A household survey using a standardized questionnaire was carried out across Tengger villages. The survey encompassed seven villages: Tosari, Wonokitri, and Podokoyo in Pasuruan Regency; Ngadisari in Probolinggo Regency; Ngadas in Malang Regency; Ranupani and Argosari in Lumajang Regency. Notably, some of these areas are situated within the BTSNP. The Tengger people, predominantly residing in the vicinity of the BTSNP and settled in the seven villages (Fig. 1), mainly inhabit areas around Mount Bromo and predominantly engage in agricultural activities (Fatjerin & Budirahayu, 2021; Kuspraningrum et al., 2020).

### Data collection

We conducted semi-structured interviews in the seven Tengger villages. Non-probability sampling was used to select targeted villages and respondents for this study. This sampling method involves choosing samples based on criteria such as the research purpose, participant availability, subjective judgment, or other non-statistical criteria rather than predetermined probabilities (Guo & Hussey, 2004). The interviews were conducted with one representative per household, selected opportunistically through purposive sampling. These interviews were held privately, without the presence of other household members or villagers. Only individuals aged ≥ 18 years were included in the study. Respondents above 18 were legally and cognitively capable of understanding the research and providing informed consent (Creswell & Creswell, 2017). The seven Tengger villages selected around the BTSNP were Tosari, Wonokitri, Podokoyo, Ngadisari, Ngadas, Ranupani, and Argosari (BTSNP, 2022). A total 641 respondents were interviewed. We used the Kuder-Richardson 20 formula (Nja et al., 2023) for the reliability test. Additionally, observations of people’s activities in the fields were made to facilitate in-depth conversations.

The questionnaire, including both closed and open-ended questions, took ~ 35 min to complete (Appendix 1). It gathered data on respondents’ attributes and sociodemographic, followed by questions about their TEK of Javanese edelweiss. Participants were asked if they could identify a picture of Javanese edelweiss, knew its name in the local language, were aware of its protected status, and knew any myths associated with it. During the interview, photographs were shown to participants (Nash et al., 2016). Follow-up questions probed the depth of their knowledge. The questionnaire also included queries about cultural values and local uses of wildlife (Archer et al., 2020). Open-ended questions about the use of Javanese edelweiss in the community aimed to elicit local knowledge. Further questions asked respondents if they recognized the species, had seen it, the year of their last sighting, location, habitat types, sighting frequency, and their perceptions of conservation status and population trends over the past decade. A map was generated based on the sighting information collected from respondents. The tool used for generating the map was QGIS 3.34.5 “Prizren.”

### Quantitative analysis

The collected data such as sociodemographic factors, recognition, sighting, perception of conservation status and population trends were analyzed using R software version 4.2.2 (R Core Team Peter Dalgaard, 2022). Variables influencing respondent recognition of edelweiss, sightings of the species, understanding of edelweiss exploitation, willingness to monitor edelweiss, and continued use despite its rarity were investigated using generalized linear mixed models (GLMMs) with a binomial error structure, as the response variables were binary (yes/no) (Archer et al., 2020). In generalized linear mixed models (GLMMs), the *p*-value is a statistical measure used to determine the significance of the estimated effects of predictor variables on the response variable. If the *p*-value is below a predetermined significance level (0.05), so there is sufficient evidence to conclude that there is a significant association between the predictor variables and the response variable (Bonovas & Piovani, 2023). Variables influencing respondents’ perceptions of species population changes and abundance were analyzed using ordinal logistic regression models with the Ordinal R package (Hirk et al., 2020). Models were fitted using the CLMM function to include random effects (Archer et al., 2020).

## Result

A total of 641 respondents completed the survey. The sociodemographic characteristics of the respondents are presented in Table 1.

**Table 1:** Demographic characteristics of respondents

Sociodemographic characteristics		Number of respondents
Sex	Male	411
	Female	230
Age	Mean age (range)	43 (18–90)
Education	No formal education	86
	Elementary school	323
	High school	214
	University	18
Residency duration	Mean (range)	41 (1–90)
Religion	Muslim	351
	Christian	7
	Hindu	255
	Buddhist	28
Occupation	Farmer	547
	Private employment	9
	Shop owner/trader	33
	Student	3
	Teacher	6
	Other	43

### Javanese edelweiss recognition and sightings

The recognition and knowledge of Javanese edelweiss in the BTSNP area were high, with 96.3% (*n* = 617) of respondents were able to recognize the plant and 73.2% (*n* = 469) reported sightings. A majority of 96.3% (*n* = 618) provided the local name for Javanese edelweiss. Respondent age significantly influenced recognition (see Appendix: Table 3) and sightings of Javanese edelweiss. Religion also influenced recognition and sightings. Individuals with higher education levels showed greater recognition of Javanese edelweiss. Additionally, the duration of residency significantly impacted sightings. Last sightings were reported in various land cover types, with secondary growth forest being the most common (52%, *n* = 110), followed by open fields (16%, *n* = 34). The majority of the last sightings (91.5%, *n* = 196) occurred in the National Park area, with a smaller proportion (8.5%, *n* = 18) in village areas (Appendix: Fig. 6).

**Fig. 2:**
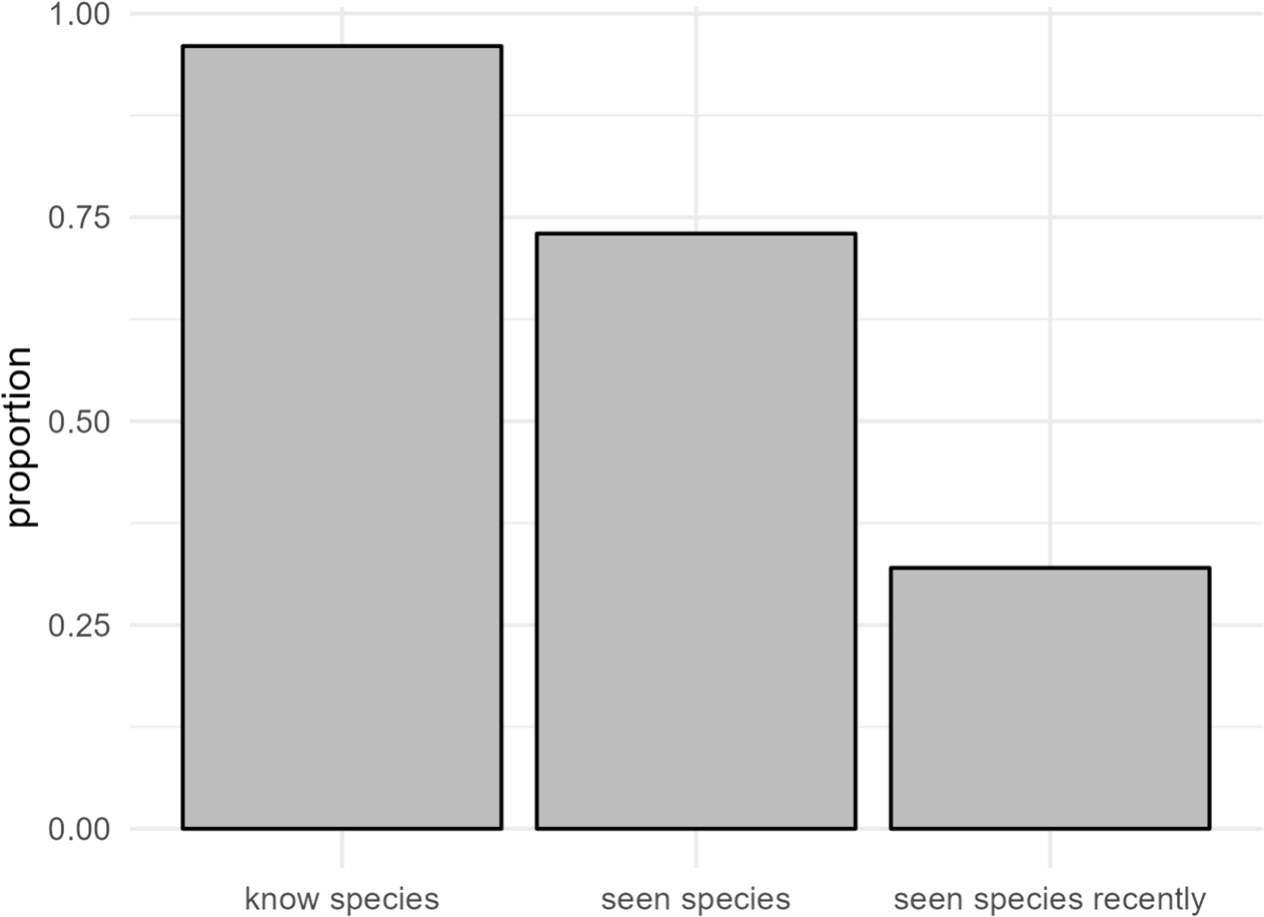
A proportion of respondents who know, seen, and reported seen recently (2021–2022) sightings of Javanese edelweiss

**Fig. 3:**
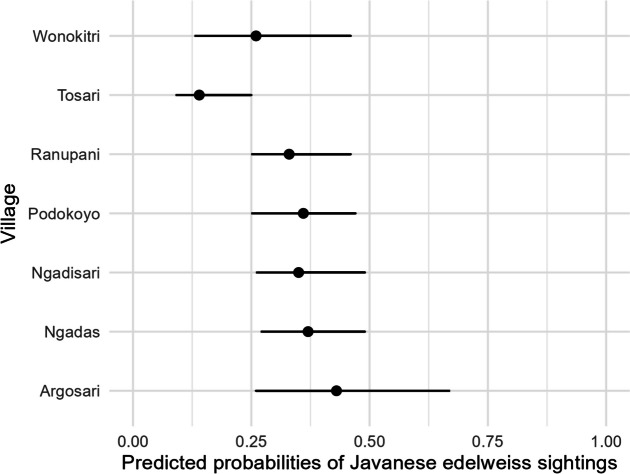
Model-predicted probabilities of recent Javanese edelweiss sightings across villages, covering the period from January 2021 to September 2022, and based on the subset of respondents who could recognize Javanese edelweiss

Significant differences were observed when comparing overall sightings to more recent sightings (2021–2022) of Javanese edelweiss (Fig. 2). A total of 32.92% (*n* = 211) of respondents reported sightings during 2021–2022. Similar to overall sightings, the village significantly influenced whether respondents had recently seen Javanese edelweiss (Appendix: Table 3), with the highest model-predicted probabilities of recent sightings in Argosari (Fig. 3). The results indicate that respondents in Argosari and Ngadas had significantly higher sighting probabilities compared to those in Tosari and Wonokitri.

### Local use and knowledge of exploitation

A majority of residents (94.7%; *n* = 607) knew that Javanese edelweiss is used in traditional ceremonies such as Karo, Unan-unan, Yadnya-Kasada, Entas-entas, and Wedding ceremony. A smaller proportion, 4.4% (*n* = 28), were aware of its commercial sale, and 1% (*n* = 6) knew of its medicinal applications. Javanese edelweiss is recognized for its medicinal properties, particularly in treating stomachaches and skin diseases (Appendix: Table 4). Among respondents who could recognize Javanese edelweiss, 82.33% (*n* = 508) continued using the plant, while 17.67% (*n* = 109) refrained from using it when it was hard to find or its population was distant from the village (Appendix: Fig. 7). Religion significantly influenced this usage pattern, with Muslim and Hinduism adherents showing a stronger inclination to continue using Javanese edelweiss (GLMM, *p* < 0.001). Muslim and Hinduism are a majority religion among Tengger people. Even though they differ in terms of religion, they bind themselves to their cultural identity as Tenggerese (Setyabudi, 2022). In the practice, Muslim Tengger and Hinduism Tengger conduct traditional ceremony and use Javanese edelweiss for this event (Appendix: Table 4).

In the subset of respondents familiar with Javanese edelweiss, 87.36% (*n* = 539) were aware of its exploitation, while 12.64% (*n* = 78) were not (Appendix: Fig. 8). Several sociodemographic factors significantly affected awareness of Javanese edelweiss exploitation, including gender, education, residency duration, and religion (Appendix: Table 3).

### Perceived Javanese edelweiss abundance and trends

**Fig. 4:**
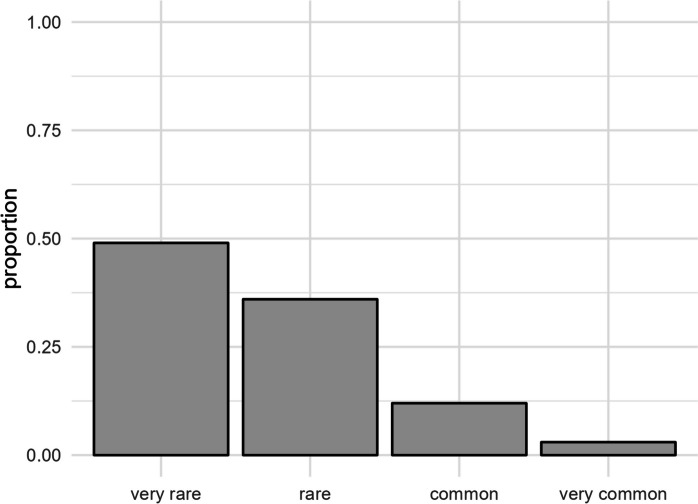
Proportion of respondents who perceived Javanese edelweiss as very rare, rare, common, or very common

Among respondents familiar with Javanese edelweiss, 48.94% (*n* = 302) perceived its population as very rare and 35.82% (*n* = 221) as rare. A smaller percentage, 12.32% (*n* = 76), perceived it as common, and only 2.92% (*n* = 18) considered it very common (Fig. 4). Village significantly affected perceptions of Javanese edelweiss abundance (Appendix: Fig. 9). The duration of residency and recent sightings in 2021–2022 also influenced these perceptions. Gender differences were significant, with male respondents more likely than females to report very rare occurrences of Javanese edelweiss (Appendix: Fig. 11).

**Fig. 5:**
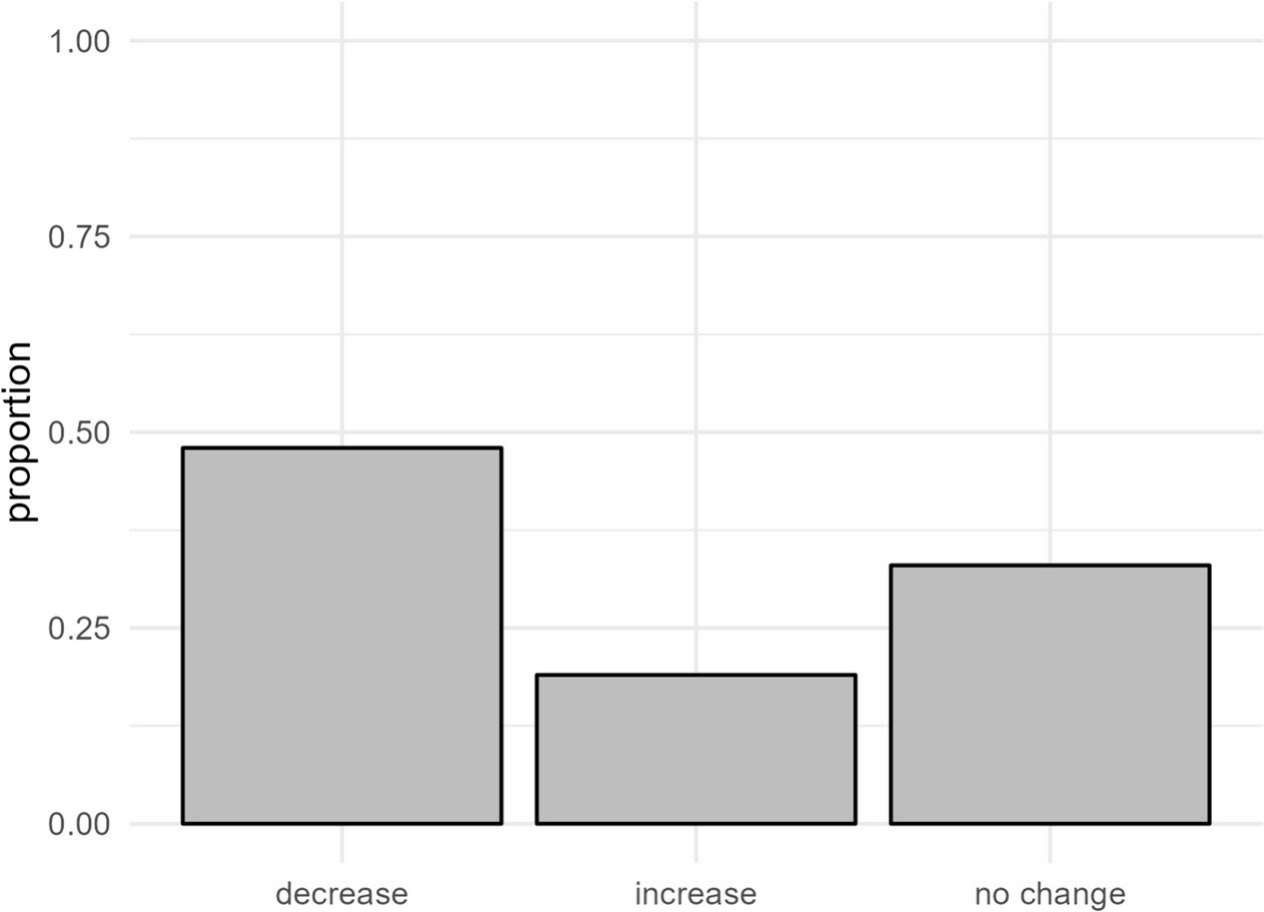
Proportion of respondents who perceived Javanese edelweiss as declining, stable, or increasing

Reports of Javanese edelweiss decline were consistent across all villages, with “decrease” being the most common response. Village, gender, and education level all significantly influenced these responses, with male respondents more frequently reporting negative population trends (Appendix: Fig. 12). Javanese edelweiss was widely perceived to be rare or very rare and reported to have declined over the past decade (Fig. 5).

### Respondent willingness to monitoring Javanese edelweiss

Among the respondents, 58.35% (*n* = 374) expressed willingness to assist in monitoring Javanese edelweiss, while 41.65% (*n* = 267) showed no willingness (Appendix: Fig. 13). Village significantly affected this willingness to help (Appendix: Fig. 14), with residents of Argosari and Wonokitri more likely to exhibit higher willingness. Gender also played a significant role, with male respondents more inclined to participate in monitoring efforts. Age, residency duration, and religion additionally influenced willingness to help.

## Discussion

This study provides crucial insights into the persistence, perceived status, population trends, and threats to species. TEK offers rapid data on the status and threats to species of conservation concern (Archer et al., 2020; Nash et al., 2016) and serves as a valuable starting point in conservation efforts, enabling the determination of species distributions at a relatively low cost (Archer et al., 2020). Our findings show that the Tengger people in the BTSNP area possess substantial recognition and knowledge of Javanese edelweiss. Additionally, a majority of respondents reported encountering this species in nature, aligning with previous studies highlighting the plant’s cultural importance among the Tengger people (Ade et al., 2021; KLHK, 2022). TEK among indigenous populations often reflects ancestral beliefs, as seen in the Tengger community’s spiritual connection with Javanese edelweiss, used in traditional ceremonies (Rahma et al., 2023).

We observed that demographic characteristics influenced locals’ ability to recognize and sight Javanese edelweiss. The study findings suggest that recognition and sightings of Javanese edelweiss vary with age. Previous research indicates that older individuals often possess greater knowledge, experience, and beliefs regarding local species (e.g., Biaou et al., 2023). Biaou et al. (2023) noted that knowledge and perception differences among age groups might stem from personal experience and exposure to the natural environment. The older age group’s familiarity with Javanese edelweiss is likely enhanced by their longer duration of living and interacting with the species.

Residency duration and religion have been identified as influencing factors in the sightings of Javanese edelweiss by the Tengger people. This observation aligns with the research of Berkes et al. (2000), which highlights that TEK is not only transmitted verbally but also acquired through experience. TEK often correlates with ecological processes influenced by religion, belief, and ethics (Berkes et al., 2000; Das et al., 2023). In some ethnicities, religious beliefs and practices can govern TEK practices. For example, it is believed among certain local ethnic groups in the Chinese plains that hunting gibbons can bring bad luck (Zhang et al., 2020). Similarly, the religion and beliefs of the Tengger people influence their TEK practices related to Javanese edelweiss, a plant that holds significant cultural and symbolic meaning for them (Pramita et al., 2013; Utomo & Heddy, 2018).

The close cultural connection of Javanese edelweiss with the Tengger people is evident in their primary use of the plant for traditional ceremonies. Most respondents (82.33%) indicated they would continue to use edelweiss for these ceremonies, even if it is difficult to find and is far from their villages. Religion emerged as a significant factor influencing the use of Javanese edelweiss. This finding is consistent with Ade et al. (2021), who noted the plant’s importance in cultural events, particularly as offerings to ancestors. The use of edelweiss is intertwined with the Tengger people, still practiced by most people in these communities.

Recent sightings suggest that Javanese edelweiss continues to persist in areas adjacent to the national park. However, most respondents considered the species to be rare or very rare in nature, and population declines were reported in every village surveyed. The infrequency of recent sightings and reports of declines indicate a recent reduction in Javanese edelweiss populations. These findings suggest that Javanese edelweiss is now relatively rare across villages adjacent to the national park. Despite its wide distribution, populations are likely small and diminishing. Consequently, there is an urgent need for conservation efforts to support this species. This necessity is reinforced by the Tengger people’s awareness of the exploitation of edelweiss. In agreement with Rahma et al. (2022), the tourism growth has contributed to the decline in Javanese edelweiss populations, exacerbated by visitor-related threats and the demand for the plant as souvenirs.

Although the overall status of Javanese edelweiss suggests that the species is now relatively rare across the region, there are noticeable geographic differences in trends and perceived status. Villages such as Argosari and Ngadas exhibit a higher probability of sightings and perceived abundance of Javanese edelweiss compared to northern areas such as Tosari and Wonokitri. This observation aligns with previous studies indicating that the distribution of Javanese edelweiss is predominantly around Mount Batok, near the Ngadas and Argosari villages (Ade et al., 2021). Utomo and Heddy (2018) reported a clustered distribution of Javanese edelweiss in savanna areas and at altitudes above 1600 m above sea level (m.a.s.l.) in Ngadas village. Conversely, respondents from Tosari and Wonokitri villages reported Javanese edelweiss as rare or very rare, indicating the highest negative population trends. This situation is likely due to the northern region being a primary tourist entry point to BTSNP, resulting in a higher threat level to Javanese edelweiss. Several studies, including those by Hall (2010) and Habibullah et al. (2016), suggest that tourism development is a contributing factor to biodiversity decline alongside climate change. Additionally, previous research noted damage to Javanese edelweiss habitats along the Mount Semeru hiking trail due to climber activities in 2018 (Amalia et al., 2019). The exploitation of Javanese edelweiss for cultural (94.69%) and economic (4.36%) needs by the community also plays a role in this trend.

Gender influences knowledge about the abundance and changes in Javanese edelweiss populations in nature. These findings are in line with studies indicating that men typically have greater knowledge and experience in interacting with nature (Chen et al., 2020; Van Der Goot et al., 2021; Zhang et al., 2020). There are notable differences in the knowledge between men and women in the Tengger community. For instance, men often engage in outdoor work, while women are more involved in household tasks, leading to men encountering Javanese edelweiss more frequently (Nunes et al., 2015). Additionally, men are usually more involved in agricultural activities, such as land clearing, which increases their likelihood of encountering various plant species (Herawati et al., 2019; Naah & Guuroh, 2017).

The results indicate that in villages such as Argosari, Wonokitri, and Ngadisari, respondents are highly willing to monitor Javanese edelweiss despite the lower recent sightings in Wonokitri. This inclination is likely due to the Tengger people’s continued need for edelweiss for traditional and economic purposes. The Tengger people utilize Javanese edelweiss for both traditional and economic purposes, reflecting its dual significance within their community. Culturally, the plant holds symbolic importance and is integrated into various traditional ceremonies and rituals (Rahma et al., 2023). These findings align with previous research, suggesting that the greater the perceived benefits by a community, the higher their willingness to engage in conservation efforts (Zhang et al., 2022). Consequently, collaboration in developing Javanese edelweiss conservation initiatives is being undertaken in these villages (BTSNP, 2022). The high levels of knowledge among the local people, their willingness to participate in conservation, and awareness of Javanese edelweiss exploitation in BTSNP can provide conservation managers with valuable insights into ecological and social issues, thereby aiding in garnering support for conservation (Bennett et al., 2017; Archer et al., 2020). Integrating TEK and local community attitudes can establish a foundation for understanding local considerations and the impact of sociodemographic factors on conservation planning processes (Archer et al., 2020). This approach views local knowledge as a legitimate and crucial part of collaborative conservation (Latulippe & Klenk, 2020).

## Conclusion

Our findings emphasize the need for enhanced and prioritized Javanese edelweiss conservation initiatives. Higher sighting frequencies, perceived abundance, and willingness to help monitor the species are evident in Ngadisari, Malang Regency, and Argosari, Lumajang Regency. Therefore, these villages should be initial focal points for conservation planning. Villagers’ willingness to collaborate with national parks underscores the value of involving communities surrounding such parks in conservation efforts. Our study highlights the critical role of TEK in providing vital information about the status and threats to Javanese edelweiss in BTSNP. The perceived decline in Javanese edelweiss populations over the past decade underscores the urgency for long-term conservation planning and the initiation of community-based monitoring efforts.

### Supplementary Information

Below is the link to the electronic supplementary material.

Supplementary file1 (DOCX 18.8 MB)

Supplementary file1 (DOCX 451)

## Data Availability

Not applicable.
