# Supplementary material for: Traditional ecological knowledge for monitoring Anaphalis javanica (DC.) Sch.Bip. (Asteraceae) in Bromo Tengger Semeru National Park, Indonesia
Source: Environ Monit Assess. 2024 Jul 9;196(8):717. doi: 10.1007/s10661-024-12869-8 (PMC11233358; doi:10.1007/s10661-024-12869-8)
Supplement: Supplementary file 1 — Supplementary file1 (DOCX 18.8 MB) [file 10661_2024_12869_MOESM1_ESM.docx]

**Traditional ecological knowledge for monitoring Javanese edelweiss in Bromo Tengger Semeru National Park, Indonesia**

Denni Susanto^12*^ (0000-0001-9531-3701), Prasetyo Nugroho^1^ (0000-0002-7275-9708), and Shinya Numata^2^ (0000-0002-4673-0485)

^1^Bachelor of Applied Science in Forest Management, Vocational College of Universitas Gadjah Mada, Yogyakarta, Indonesia

^2^Department of Tourism Science, Tokyo Metropolitan University, Tokyo, Japan

^*^Corresponding author (denni.s@ugm.ac.id)

Questionnaire

Date ________ Interviewer ___________ Village ___________ Lat / Lon______________

We are researchers from Universitas Gadjah Mada and Tokyo Metropolitan University. We want to know more about village and its surroundings, so I hope you can help provide data in the form of information to assist us in knowing the customs, usage of *Anaphalis javanica* or better known with “*Tanalayu*”, and also environmental changes that affect it. This survey is anonymous and all the information you provide will only be used for research and analysis. We will not hand over and share your details with third parties.

**1. Are you willing to participate in this survey?** □ Yes □ Unwilling

**2. Basic information of the interviewee**

Age ______ Gender (M / F)_____ Education

Ethnicity (Tengger / Javanese / other)_______

Length of residency _________years

Religion (Islam / Protestant / Catholic / Hindu / Budha / Confusianism / other :

Occupation __________________________________ Retired? □ Yes □ No

**3. Knowledge of Edelweiss**

| 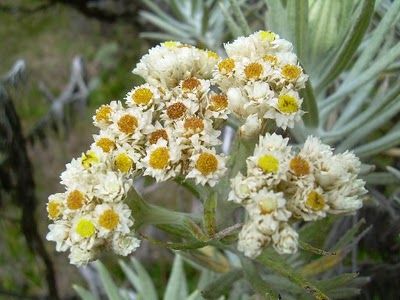 | 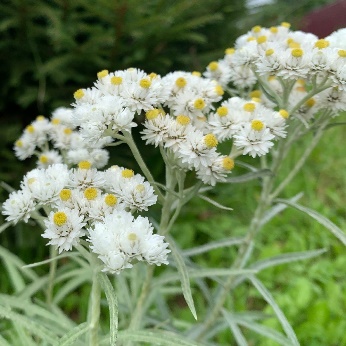 | 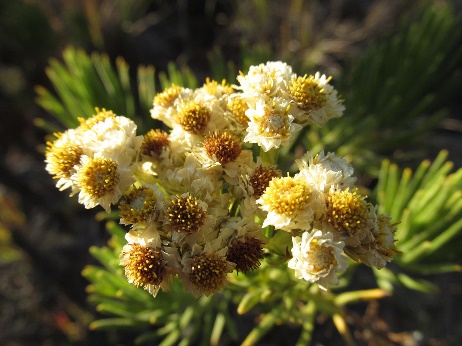 |
| --- | --- | --- |
| *Anaphalis javanica*  **Figure 1** | *Anaphalis longifolia*  **Figure 2** | *Anaphalis viscida*  **Figure 3** |

(*Anaphalis javanica*) Do you know what this plant is? □Yes □No

(*Anaphalis longifolia*) Do you know what this plant is? □Yes □No

(*Anaphalis vicida*) Do you know what this plant is? □Yes □No

If YES, describe local name / size / appearance ___________________________________

If NO, do you know what “edelweiss” is? Describe ________________________________

If NO, do you know what “(*tanalayu*)” is? Describe _______________________________

How do you know this plant? _____________________________________________________

Have you seen this plant? □Yes □No □ Don’t Know

Have you seen this plant in the past 12 months? □Yes □No

If YES, *when* was the most recent time that you have seen it? _________________________

If YES, *where* have you seen it (show the map)?____________________________________


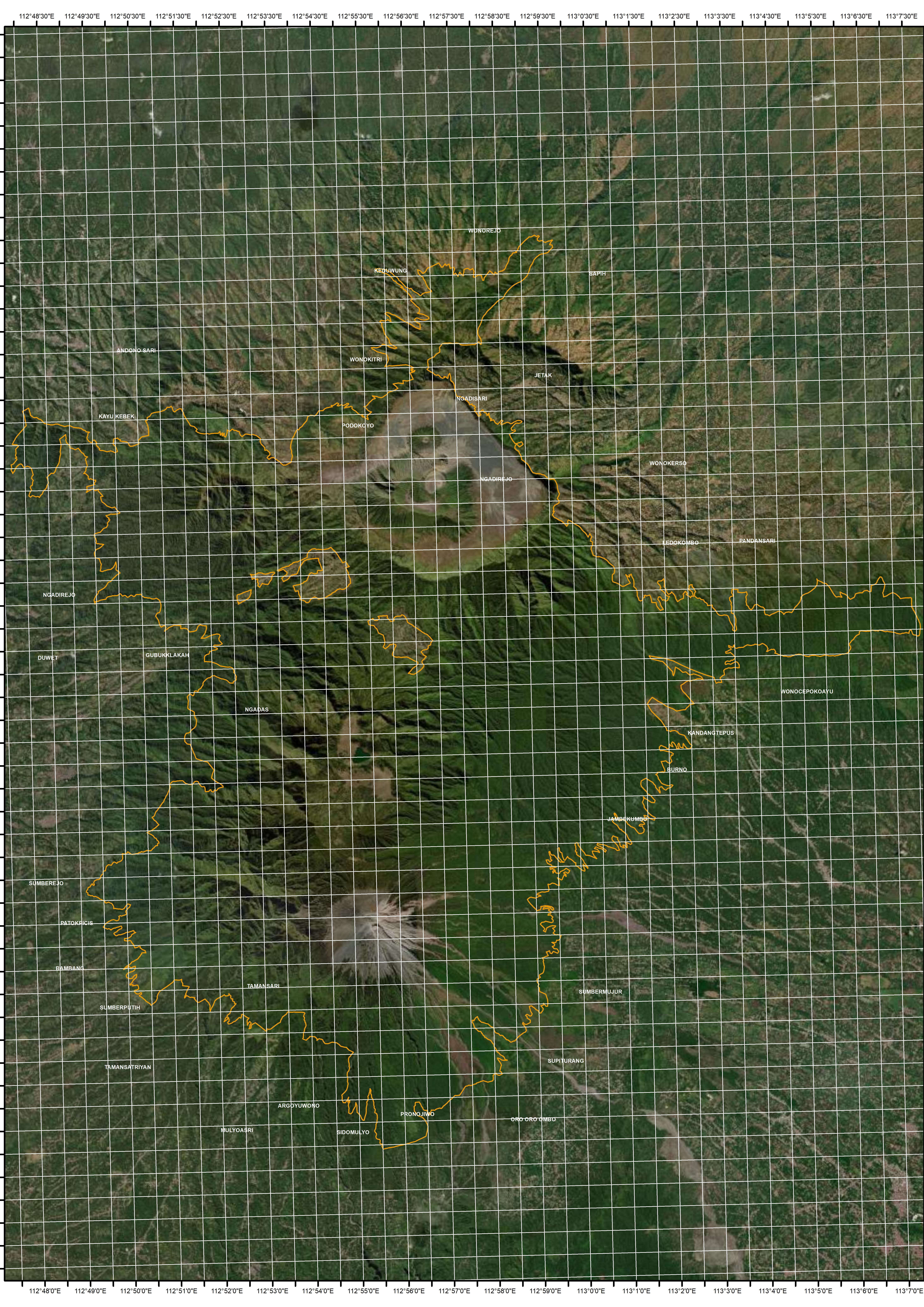


Can edelweiss be used for anything? □Yes □No □ Don’t Know

If YES, what? (describe) ______________________________________________________

If NO, Why do people not exploit edelwiss?

Do you know Edelweiss is protected plant? □Yes □No □Don’t Know

How frequently do you see this plant?

□Daily □Weekly □Monthly □Yearly □Less than yearly □Other

Over the past ten years, do you think the number of this plant has changed?

□Yes □No □Don’t Know

If yes, please state how the numbers of this plant has changed?

□Increase □Decrease □Not sure________

How common or uncommon do you think this plant?

□Very rare □Rare □Common □Very common

**4. Conservation Edelweiss**

Have you ever heard of edelweiss being exploited?

□Yes □No □ Don’t Know

If YES, How often does this happen? ____________________________________________

Is still happen now? ____________________________________________________

Do you know what edelweiss is exploited for? ____________________________

Do you hope edelweiss will still be around the village?

□Yes □No □ Don’t Know

Would you still use edelweiss if it was farther away from the village and/or the population was decreasing? □Yes □No

And finally, how willing or unwilling are you to help scientists monitor this plant in this area?

□Yes □No

Thanks for your help.
