# Supplementary material for: Traditional ecological knowledge for monitoring Anaphalis javanica (DC.) Sch.Bip. (Asteraceae) in Bromo Tengger Semeru National Park, Indonesia
Source: Environ Monit Assess. 2024 Jul 9;196(8):717. doi: 10.1007/s10661-024-12869-8 (PMC11233358; doi:10.1007/s10661-024-12869-8)
Supplement: Supplementary file 2 — Supplementary file1 (DOCX 451) [file 10661_2024_12869_MOESM2_ESM.docx]

**Traditional ecological knowledge for monitoring Javanese edelweiss in Bromo Tengger Semeru National Park, Indonesia**

Denni Susanto^12*^ (0000-0001-9531-3701), Prasetyo Nugroho^1^ (0000-0002-7275-9708), and Shinya Numata^2^ (0000-0002-4673-0485)

^1^Bachelor of Applied Science in Forest Management, Vocational College of Universitas Gadjah Mada, Yogyakarta, Indonesia

^2^Department of Tourism Science, Tokyo Metropolitan University, Tokyo, Japan

^*^Corresponding author (denni.s@ugm.ac.id)

**Tabel 2** Description of predictor variables selected a priori and used in models

| Variable Predictor | Response variable for questions on: | | | | | | | | Data format | Descriotion and hypotheses | Variable type | Literature |
| --- | --- | --- | --- | --- | --- | --- | --- | --- | --- | --- | --- | --- |
|  | **Javanese edelweiss recognition (picture)** | **Javanese edelweiss sightings** | **Recent Javanese edelweiss sightings** | **Know Javanese edelweiss exploitation** | **Willingness to help monitoring** | **Keep using edelweiss** | **Perceived Javanese edelweiss abundance** | **Perceived Javanese edelweiss trends** |  |  |  |  |
| Gender | ✓ | ✓ | ✓ | ✓ | ✓ | ✓ | ✓ | ✓ | Continuous (years) | Male gender show a higher knowledge and experience in nature interaction context because male usually does the largescale of agricultural activities. | Fixed | (Herawati et al. 2019; Chen et al. 2020; Zhang et al. 2020; Van Der Goot et al. 2021). |
| Age | ✓ | ✓ | ✓ | ✓ | ✓ | ✓ | ✓ | ✓ | Categorical dichotomous (male or female) | Older participants have more time to learn to recognise and encounter species in the nature, so that the older participants more have a knowledge. | Fixed | (Chen et al. 2020; Van Der Goot et al. 2021). |
| Education | ✓ | ✓ | ✓ | ✓ | ✓ | ✓ | ✓ | ✓ | Categorical (ordinal - level of education) | Education level could also affect the people’s perception of nature or species. | Fixed | (Hicks and Cinner 2014). |
| Residency duration | ✓ | ✓ | ✓ | ✓ | ✓ | ✓ | ✓ | ✓ | Continuous (years) | The participants who stay long in the village, will have high experience of encounter with animals, and t o form knowledge about these species. | Fixed | (Turvey et al. 2017; Okui et al. 2021). |
| Religion | ✓ | ✓ | ✓ | ✓ | ✓ | ✓ | ✓ | ✓ | Categorical (nominal) | As a knowledge–practice–belief complex, traditional ecological knowledge includes the religious traditions of a society. | Fixed | (Gann et al. 2019). |
| Villages | ✓ | ✓ | ✓ | ✓ | ✓ | ✓ | ✓ | ✓ | Categorical (nominal) | Villages may be subject to different natural resource management strategies. | Fixed | (Nash et al. 2016). |
| Enumerator | ✓ | ✓ | ✓ | ✓ | ✓ | ✓ | ✓ | ✓ | Categorical (nominal) | Interviews can be subject to interviewer bias, with the potential for questions to be  answered differently depending on who is asking the questions. This was therefore included in the models to check for bias. | Random | (Newing 2011). |

**Tabel 3** Summary of generalized linear mixed model (GLMM) and cumulative link mixed model (CLMM) result

|  | Full model | Significant variables | SE | p-value |
| --- | --- | --- | --- | --- |
| GLMM | Model 1: Edelweiss recognized ~ village + age + gender + education + residency duration + religion (1\|enumerator) | Village | 1.523e-01 | 0.640583 |
|  |  | Age | 3.822e-02 | 0.028937 * |
|  |  | Gender | 2.020e+06 | 0.999987 |
|  |  | Education | 3.664e-01 | 0.000349 *** |
|  |  | Residency duration | 3.494e-02 | 0.556179 |
|  |  | Religion | 2.364e-01 | 0.020145 * |
| GLMM | Model 2: Edelweiss sighting ~ village + age + gender + education + residency duration + religion (1\|enumerator) | Village | 0.09583 | 0.28882 |
|  |  | Age | 0.01993 | 0.00139 ** |
|  |  | Gender | 0.34513 | < 2e-16 *** |
|  |  | Education | 0.16124 | 0.09958 |
|  |  | Residency duration | 0.01822 | 3.45e-09 *** |
|  |  | Religion | 0.12265 | 4.22e-07 *** |
| GLMM | Model 3: Edelweiss recent sighting ~ village + age + gender + education + residency duration + religion (1\|enumerator) | Village | 0.07610 | 0.005948 ** |
|  |  | Age | 0.02509 | 0.013389 * |
|  |  | Gender | 1801.29573 | 0.990828 |
|  |  | Education | 0.15993 | 0.483430 |
|  |  | Residency duration | 0.02439 | 0.000858 *** |
|  |  | Religion | 0.08460 | 2.43e-07 *** |
| GLMM | Model 4: Know edelweiss exploitation ~ village + age + gender + education + residency duration + religion (1\|enumerator) | Village | 0.082164 | 0.98584 |
|  |  | Age | 0.017582 | 0.80211 |
|  |  | Gender | 0.396002 | 1.03e-15 *** |
|  |  | Education | 0.167532 | 0.00937 ** |
|  |  | Residency duration | 0.015189 | 0.00770 ** |
|  |  | Religion | 0.110692 | 0.02880 * |
| GLMM | Model 5: Willingness edelweiss monitoring ~ village + age + gender + education + residency duration + religion (1\|enumerator) | Village | 0.08552 | 9.69e-13 *** |
|  |  | Age | 0.01706 | 0.000190 *** |
|  |  | Gender | 0.21620 | 0.000106 *** |
|  |  | Education | 0.15695 | 0.000791 *** |
|  |  | Residency duration | 0.01622 | 0.000177 *** |
|  |  | Religion | 0.08050 | 0.000501 *** |
| GLMM | Model 6: Keep using edelweiss ~ village + age + gender + education + residency duration + religion (1\|enumerator) | Village | 0.090029 | 0.889 |
|  |  | Age | 0.015594 | 0.868 |
|  |  | Gender | 0.241808 | 0.936 |
|  |  | Education | 0.147917 | 0.675 |
|  |  | Residency duration | 0.014095 | 0.247 |
|  |  | Religion | 0.109314 | 8.26e-06 *** |
| CLMM | Model 7: Edelweiss population trends ~ village + age + gender + education + residency duration + religion (1\|enumerator) | Village | 0.075288 | 0.03273 * |
|  |  | Age | 0.013307 | 0.63597 |
|  |  | Gender | 0.236674 | < 2e-16 *** |
|  |  | Education | 0.124639 | 0.00297 ** |
|  |  | Residency duration | 0.012110 | 0.54539 |
|  |  | Religion | 0.074331 | 0.10215 |
| CLMM | Model 8: Perceived edelweiss abundance ~ village + age + gender + education + residency duration + religion (1\|enumerator) | Village | 0.06263 | 2e-07 *** |
|  |  | Age | 0.01326 | 0.096949 . |
|  |  | Gender | 0.17608 | 0.000972 *** |
|  |  | Education | 0.11206 | 0.037166 * |
|  |  | Residency duration | 0.01225 | 0.013150 * |
|  |  | Religion | 0.06060 | 0.270728 |

| Use | Total responden | Information |
| --- | --- | --- |
| Traditional ceremony | 607 | Karo, Unan-unan, Yadnya-Kasada, Entas-entas, and Wedding ceremony |
| Economic | 28 | Selling for souvenir |
| Medicine | 6 | stomachache and skin diseases |

**Table 4** Respondents’ knowledge based on edelweiss usage

**
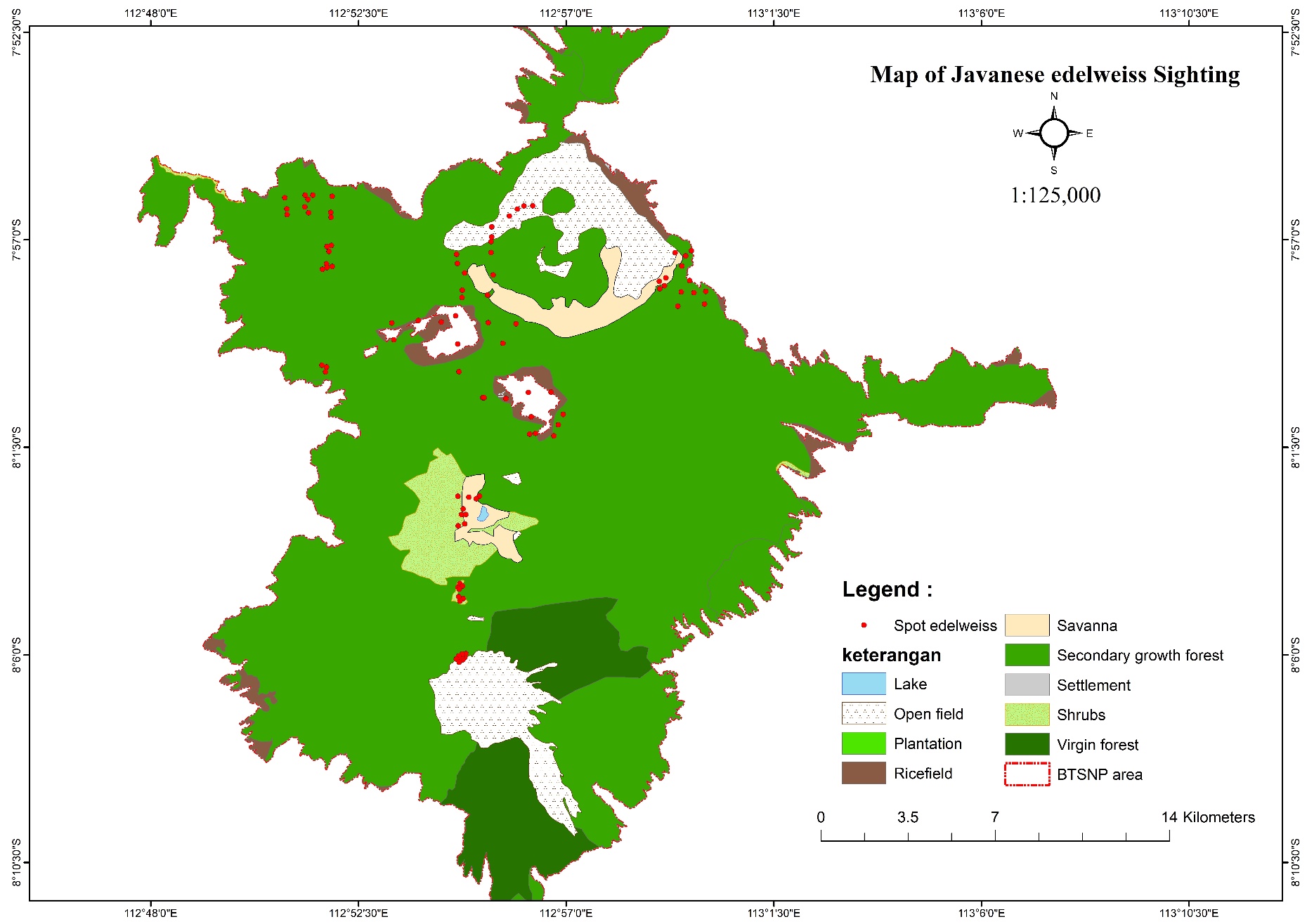
**

**Fig. 6** Map of Javanese edelweiss sighting (2021-2022)

**Fig. 7** Bar chart of keep using Javanese edelweiss

**Fig. 8** Bar chart of Javanese edelweiss exploitation knowledge

0.75

0.5

0.25

**Fig. 9** Bar chart of perceived abundance of Javanese edelweiss based on village

0.25

0.5

0.75

**Fig. 10** Bar chart of perceived trends of Javanese edelweiss based on village

0.75

0.25

0

1

0.5

**Fig. 11** Bar chart of perceived abundance of Javanese edelweiss based on gender

0

1

0.75

0.5

0.25

**Fig. 12** Bar chart of perceived trends of Javanese edelweiss based on gender

**Fig. 13** Bar chart of willingness to help monitoring of Javanese edelweiss

0.75

0.25

0.5

**Fig. 14** Bar chart of willingness to help monitoring of Javanese edelweiss based on village
